# Comparison of major, minor and junctional circumsporozoite protein epitopes for malaria vaccine design

**DOI:** 10.1038/s41541-025-01264-0

**Published:** 2025-10-03

**Authors:** Emma Ryan, Dallas Brown, William Harrison, Shelby Foor, Kutub Ashraf, Jessica S. Bolton, Yevel Flores-Garcia, Randall S. MacGill, Emily Locke, Elke Bergmann-Leitner, Alison E. Roth, Paul M. Robben, Gary Matyas, Lorraine Soisson, Robin Miller, Adrian H. Batchelor, Fidel Zavala, Sheetij Dutta

**Affiliations:** 1 Structural Vaccinology Laboratory, Biologics Research and Development Branch, Center for Infectious Diseases Research, Walter Reed Army Institute of Research, Silver Spring, MD USA; 2 Integrated Pathogen Therapeutics Department, Viral Disease Program, Walter Reed Army Institute of Research, Silver Spring, MD, USA; 3 Immunology Core, Biologics Research and Development Branch, Walter Reed Army Institute of Research, Silver Spring, MD USA; 4 Molecular Microbiology and Immunology, Johns Hopkins University, Baltimore, MD USA; 5 Center for Vaccine Innovation and Access, PATH, Washington, DC USA; 6 Biologics Research and Development Branch, Center for Infectious Diseases Research, Walter Reed Army Institute of Research, Silver Spring, MD USA; 7 Antigen and Adjuvant Research Branch, Military HIV Research Program, Center for Infectious Diseases Research, Walter Reed Army Institute of Research, Silver Spring, MD USA; 8 United States Agency for International Development, Washington, DC USA; 9 Present Address: Department of Molecular Biosciences, The University of Texas at Austin, Austin, TX USA

**Keywords:** Immunology, Microbiology, Diseases

## Abstract

Currently approved malaria vaccines (RTS,S/AS01 and R21/Matrix-M) contain the tetrapeptide major repeats (19x NPNA) and C-terminal domain of the circumsporozoite protein of *Plasmodium falciparum*. Incorporating the junctional (NPDP) and minor repeat (NPNV) epitope targeted by protective human monoclonal antibodies into immunogens is hypothesized to improve vaccine efficacy. However, comparisons of such candidates have yielded contradictory results due to inter-study differences. Tobacco mosaic virus (TMV) capsid virus-like particles displaying the minor repeat, junctional, and major repeat epitopes were compared in an intravenous challenge model. Despite high cross-reactivity and in vitro inhibition, minor repeat candidates did not confer sterile protection in vivo. Constructs displaying major repeats NPNAx20, NPNAx5, and a junctional+minor repeat epitope induced sterile protection. Head-to-head comparisons of selected TMV vaccines and RTS,S revealed equivalent in vivo liver burden reduction. TMV-NPNAx20 was selected for clinical-grade antigen manufacture based on its equivalent reduction in parasite burden at lower antibody concentrations.

## Introduction

*Plasmodium falciparum* malaria remains a significant global public health threat^1^. Growing insecticide resistance and the reduced efficacy of rapid diagnostic tests and frontline antimalarials risk the reversal of global progress on malaria control^2,3^. Infection begins after a mosquito carrying *P. falciparum* parasites deposits ~100 sporozoites into the human dermis during a blood meal, which the parasite navigates through to reach the liver and infect hepatocytes^3^. Each sporozoite is densely covered with the circumsporozoite protein (CSP), which is composed of the N- and C-terminal regions joined by a structurally disordered array of repeating tetrapeptide motifs^4–6^. It has been hypothesized that hepatocyte invasion requires a proteolytic processing event that cleaves the N-terminus and exposes the C-terminus, allowing sporozoites to interact with surface-expressed factors on hepatocytes^7–9^.

The function of the CSP repeat region has remained largely unknown, but the CSP of all *P. falciparum* strains contains three conserved elements: 1x leading NPDP (junctional), ~38x NPNA (major), and 3-4x NPNV (minor) tetrapeptide repeats^10^. CSP is the basis for two World Health Organization (WHO)-recommended malaria vaccines, RTS,S adjuvanted with AS01 and R21 adjuvanted with Matrix-M^11^. Both vaccines contain an identical core immunogen comprised of 19x NPNA repeats and the C-terminus of CSP expressed on the hepatitis B *S* antigen particle. Three-dose regimens of both vaccines induce ~50-80% sterile protection against experimental mosquito bite challenge in malaria-naïve volunteers. Among naturally exposed target populations, the three-dose primary series followed by an annual booster confers ~50–75% protection from clinical malaria over 12 months in young African children^12–20^. Although antibody responses are short lived, widespread implementation of RTS,S and R21 in moderate- to high-transmission settings (the current WHO recommendation) is expected to save thousands of lives in Africa in the near term. Malaria elimination, however, may require next-generation vaccines that provide multi-year protection against infection and transmission across different age groups in endemic areas, as well as approval for travelers and the military.

In comparison to SARS-CoV-2 (~3.6 µg/mL), *Haemophilus influenzae* type b (0.15 µg/mL), and hepatitis B (1 µg/mL)^21^, the vaccine-induced antibody concentration required to prevent infection is much higher for malaria vaccines (>100 µg/ml for RTS,S)^22,23^. Structure-based design can be used to improve vaccines by focusing responses to the most protective epitopes and discouraging the induction of bystander antibodies^24,25^. Work in the years since the design of the RTS,S antigen has delineated the molecular attributes recapitulated by some of the most potently protective anti-CSP monoclonal antibodies (mAbs), which are now being leveraged to design next-generation candidates. These antibodies are predominantly produced by the *VH3-33*/*VK1-5* gene pairing, bind to the repeat region in type I β- and/or pseudo Asx 3_10_ turn conformations, and are stabilized by Fab-Fab homotypic contacts^26–30^. Two additional protective epitopes flanking the N-terminus and major repeats have also since been identified, termed the junctional (NPDPNANPNVDPNAN^31^) and the minor repeat epitope (NANPNVDPNANPNVD^31,32^). MAbs against CSP demonstrate varying binding promiscuities for these three epitopes, with important implications for immunity^28^. While antibodies with a strong preference for the major repeats and ancillary activity for the junctional region were found to be most inhibitory^29^, a murine study using chimeric *Plasmodium berghei* parasites expressing only the minor repeat, junctional, or major repeat epitopes of *P. falciparum* established that mAbs may not rely on cross-reactivity to neutralize sporozoites^33^.

MAbs against the major repeat (mAbs 317, 311, and MAM01), junctional (mAb CIS43), and minor repeat (mAb L9) epitopes can protect against controlled *Plasmodium* infection in preclinical models and humans^31–36^. The most clinically advanced candidate mAb L9, selected on the basis of its superiority over mAbs CIS43 and 317 in a murine challenge model^32^, was shown to protect humans against controlled and naturally transmitted *P. falcipaum* infection at serum concentrations as low as 10 µg/mL^36^. MAb L9 has singular attributes that differ from the majority of identified CSP mAbs, which render it an interesting target for vaccine design, including its baseline specificity for the NPNV minor repeat and ability to react with long NPNA major repeats via evolved Fab-Fab contacts that increase its overall avidity^32,37^.

The majority of next-generation CSP vaccine efforts rely on displaying CSP mAb epitopes on virus-like particles (VLPs). A multitude of VLPs, including the woodchuck hepatitis virus core antigen^38^, E2 protein of chikungunya virus^39^, Q-beta bacteriophage^40,41^, *Helicobacter pylori* apoferritin^42^, tobacco mosaic virus (TMV) coat protein^43,44^, and cucumber mosaic virus coat protein^45^, are effective in augmenting the immunogenicity of small CSP repeat epitopes. However, differences in epitope density, cadence, context, and valency, as well as selected carrier protein, adjuvant, and protection model across studies have confounded the down-selection of the most potent CSP epitopes to be used for immunogen design.

The Walter Reed Army Institute of Research (WRAIR) has previously shown that TMV-based malaria vaccine candidates displaying a truncated (NPNAx5; T5) or extended (NPNAx20; T20) version of the major repeats or a junctional+minor repeat epitope (T51) were more protective than a soluble, nearly full-length CSP (FL-CSP) vaccine in a mouse transgenic parasite challenge model^43,44^. We now report the head-to-head assessment of minor repeat-, junctional+minor repeat-, and major repeat-focused TMV vaccine candidates in two independent murine challenge models. We also performed fine dissection of the serological profile of protective CSP vaccines using functional assays and selected a TMV-based vaccine candidate for current Good Manufacturing Practice (cGMP) manufacture.

## Results

### Design and purification of novel minor repeat VLPs

**Fig. 1:**
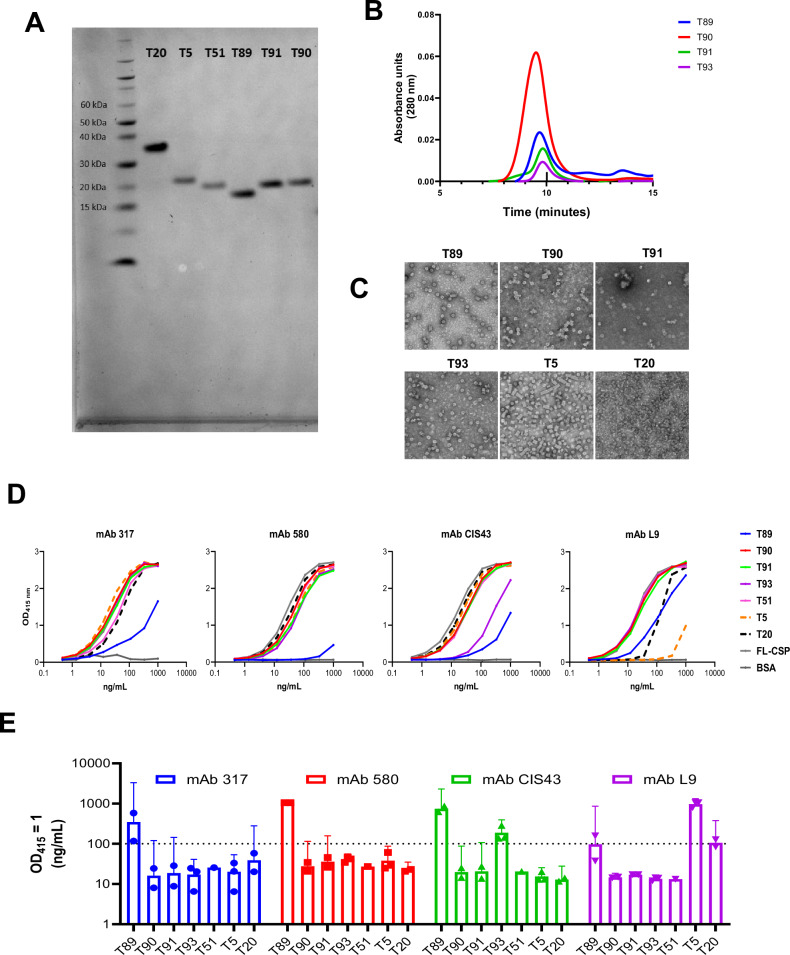
Biophysical and biochemical characteristics of immunogens. **A** Reducing SDS-PAGE analysis of purified antigens. **B** Representative HPLC-SEC analysis showing elution volumes of TMV VLPs (~*10* *min*). **C** Negative-stain electron microscopy of purified products demonstrating particle morphology. **D**, **E** ELISA curves of CSP mAb 317, mAb 580, mAb CIS43, and mAb L9 binding to minor repeat-, junctional+minor repeat-, and major repeat-focused VLPs and the ng/mL of mAb required to reach OD_415 nm_ = 1 (described as antigenicity). BSA was used as a negative control, and FL-CSP as a positive control. Mean values with 95% confidence intervals are plotted.

The mAb L9 epitope in multiple cadences and valencies was displayed on the exposed loop of the TMV VLP platform (Table 1)^32^. T89 displayed the minimal L9 epitope, while longer versions T90 and T91 were designed to accommodate homotypic interactions. A non-native NPNV-enriched T93 was designed to reduce the induction of poorly neutralizing minor repeat antibodies like mAb F10^37^. All VLPs were pure and homogenous as assessed by SDS-PAGE and SEC-HPLC, with the chromatograms indicating that the majority the protein eluted as a VLP (Fig. 1A, B, Supplementary Fig. 6). Negative-stain electron microscopy confirmed TMV disks with ~20-nm diameter and a central pore, although some constructs, such as T5 and T89, also showed disk stacks (Fig. 1C).

**Table 1:** Epitopes displayed by each antigen used across studies

Vaccine	Region	Epitope
T89	Minor	NPNVDPNANPNV
T90		DPNANPNVDPNANPNVDPNA
T91		NPNVDPNANPNVDPNANPNVDPNA
T51	Junctional+minor	NPDPNANPNVDPNANPNVDPNANPNA
T93	Non-native minor	NPNVNPNVNPNVNPNVDPNA
T5	Short major	NPNAx5
T20	Extended major	NPNAx20

The antigenic profile of each VLP was determined by an ELISA using a suite of CSP mAbs: mAb 317 (high-affinity major repeat-specific)^46^, 580 (low-affinity major repeat-specific)^47^, CIS43 (junctional-specific)^31^, and L9 (minor repeat-specific)^32^ (Fig. 1D). The shortest minor repeat VLP (T89) was poorly antigenic, requiring >100 ng/mL mAb to reach OD_415 nm_ = 1 (Fig. 1E). All other VLPs were potently bound by mAbs 317, 580, and CIS43, with the exception of T93’s binding by mAb CIS43. MAb L9 was more selective, demonstrating reduced binding to the major repeat VLPs (T5 and T20), which is consistent with its described behavior^32,37^. These results confirmed the TMV platform’s ability to display high-fidelity, conformationally active CSP epitopes.

### Minor repeat (T89, T90, T91, and T93) *vs*. major repeat (T5 and T20) constructs

**Fig. 2:**
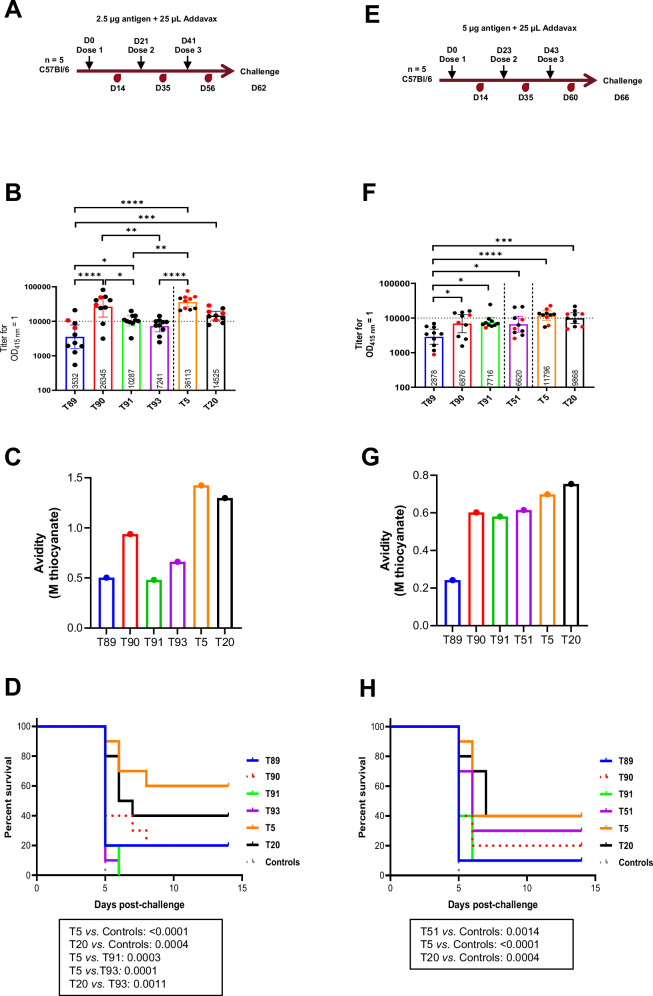
WRAIR down-selection challenge studies indicate that minor repeat- and junctional+minor repeat-focused vaccines do not improve upon major repeat-focused vaccines. Addavax-adjuvanted vaccines were administered to C57Bl/6 mice (*n* = 10). **A**–**D** First study (three 2.5 µg antigen doses); **E**–**H** second study (three 5 µg antigen doses). **A**, **E** Schematic of study outline showing vaccination and sera collection time-points. Challenge via intravenous administration of transgenic *PbPf* sporozoites occurred 3 weeks post-third dose (3WP3). **B**, **F** Geometric mean ELISA titer (OD_415 nm_ = 1) with 95% confidence intervals using 2WP3 sera. Red symbols denote protected mice. **C**, **G** FL-CSP avidity (mean of duplicate wells) measured using pooled group sera at 2WP3. Plotted values are the molarity of sodium thiocyanate required to reduce the maximal OD_415 nm_ by 50%. **D**, **H** Survival curves after intravenous challenge with 3000 transgenic *PbPf* parasites.

In the first WRAIR challenge study, C57Bl/6 mice (*n* = 10) received three 2.5 µg doses of VLPs adjuvanted in Addavax at 3-week intervals (Fig. 2A). A full-length CSP (FL-CSP) ELISA with sera obtained 2 weeks post-third (2WP3) vaccination (Fig. 2B) revealed low (<10,000) titers in the T89 group, in accordance with its low antigenicity; the other minor repeat VLPs, excluding T93, and the major repeat candidates demonstrated significantly higher immunogenicity than T89. While the titer of the T5-vaccinated group was highest, it did not significantly differ from the T20-vaccinated group’s titer. An avidity ELISA revealed that the avidities of antibodies induced by T5 and T20 trended higher than minor repeat VLPs’ (Fig. 2C). WRAIR’s transgenic intravenous parasite challenge with 3000 sporozoites 2 weeks post-third (2WP3) dose infected all naïve controls (Fig. 2D). T5 (60%; *p* < 0.0001) and T20 (40%; *p* = 0.0004) showed improved survival over the control group, while the minor repeat VLPs T89, T90, T91, and T93 all showed non-significant survival compared to the control group (*p* > 0.05) (Fig. 2D).

### Minor repeat (T89, T90, and T91) *vs*. major repeat (T5 and T20) *vs*. junctional+minor repeat (T51) constructs

In the second WRAIR challenge study, C57Bl/6 mice (*n* = 10) received three 5 µg doses of VLPs adjuvanted in Addavax at three-week intervals. The higher antigen doses were intended to compensate for the lower immunogenicity of some minor repeat vaccines. The junctional+minor repeat VLP, T51, was included in this study in place of T93 (Fig. 2E). Despite a higher antigen dose, the immunogenicity of the TMV VLPs at a 5 µg dose was generally lower than the immunogencity observed at a 2.5 µg dose, as has also been observed for RTS,S in mouse models^48,49^. This may reflect a feedback inhibition of antibody boosting due to circulating CSP antibodies^50^. T89’s immunogenicity was again found to be low, with all other minor repeat groups showing higher immunogenicity (Fig. 2F). As before, the titer of the T5 recipient group was highest, but not significantly higher than the T20 recipient group’s. Assessing the avidity of 2WP3 serum pools using FL-CSP again showed a weak trend favoring T5 and T20 (Fig. 2G). Challenge with transgenic *PbPf* sporozoites at 2WP3 showed no significant increase in survival for the minor repeat VLPs T89, T90, and T91, but T5 (40%; *p* < 0.0001), T20 (40%; *p* = 0.0004) and T51 (30%; *p* = 0.0014) all had significantly improved survival compared to the controls (Fig. 2H). These two experiments indicated that minor repeat VLPs adjuvanted with Addavax were less protective than major and junctional+minor repeat constructs in WRAIR’s sterile protection model. When comparing sterilely protected mice (red symbols) with non-protected mice (black symbols) in both studies, a high FL-CSP titer was not found to be necessary for protection (Fig. 2B, F).

### In vitro neutralization by immune sera

**Fig. 3:**
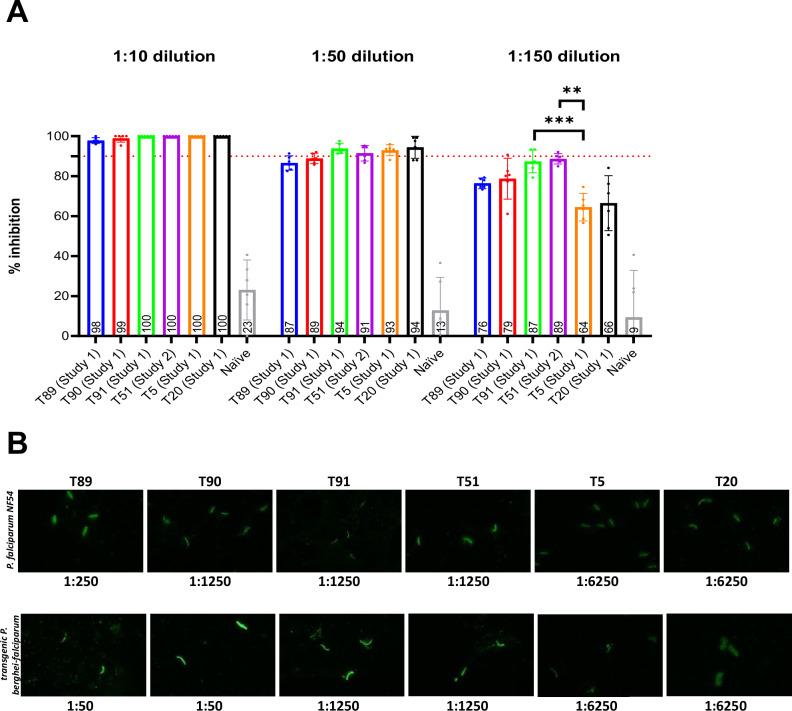
In vitro characterization of vaccine-induced polyclonal sera: inhibition of liver stage development assay (ILSDA) and immunofluorescence assay (IFA). **A** ILSDA using Addavax-adjuvanted sera conducted at 1:10, 1:50, and 1:150 serum dilutions. Mean values with 95% confidence intervals are plotted. While all groups retained significantly higher inhibition compared to naïve sera at a 1:150 dilution, p values for only inter-epitope (*i.e*. major *vs*. minor and junctional+minor repeat) differences in inhibition are indicated. **B** IFA using Addavax-adjuvanted sera against fixed *P. falciparum* NF54 sporozoites (top) or fixed transgenic *PbPf* sporozoites (bottom). The numbers below each panel represent end-point titers.

The invasion of *P. falciparum* sporozoites into cryopreserved primary human hepatocytes was measured, with mAbs CIS43, 317 and 2A10 serving as positive controls in an inhibition of liver stage development assay (ILSDA) (Supplementary Fig. 1). Immune sera from WRAIR’s in vivo studies at low dilutions (1:10 and 1:50) affected >80% inhibition in all VLP groups, compared to the ~20% inhibition affected by naïve controls (Fig. 3A). At a higher serum dilution (1:150), the inhibitions of T90, T91, and T51 sera remained high, while the protection induced by the major repeat VLPs T5 and T20 were significantly lower. An immune-fluoresence assay (IFA) confirmed that the polyclonal antibodies induced by all constructs bound to fixed transgenic and native *P. falciparum* sporozoites, confirming that the poor protection of the minor repeat constructs was not due to the inability of their antibodies to recognize CSP on the transgenic sporozoite surface. The major repeat VLPs T5 and T20 showed the highest IFA titers (highest serum dilution that gave a positive signal) (Fig. 3B). These results indicated a discrepancy between the in vivo sterile protection (highest for major repeat vaccines) and in vitro ILSDA readouts (highest for minor repeat vaccines). In determining which vaccines would progress into subsequent studies, we prioritized in vivo efficacy over in vitro readouts.

### Major repeat (T5 and T20) *vs*. junctional+minor repeat (T51) *vs*. RTS,S

The Johns Hopkins University (JHU) mouse challenge model allows for the direct comparison of CSP vaccine candidates to RTS,S/AS01 using quantitative liver parasite burden as the assay readout^49^. WRAIR’s first-generation, soluble, full-length CSP antigen, FMP013, formulated in clinical-grade ALFQ adjuvant^51^, was found to be less immunogenic and less potent than RTS,S/AS01, confirming the model’s ability to discriminate between the efficacy of CSP vaccines (Supplementary Fig. 2).

**Fig. 4:**
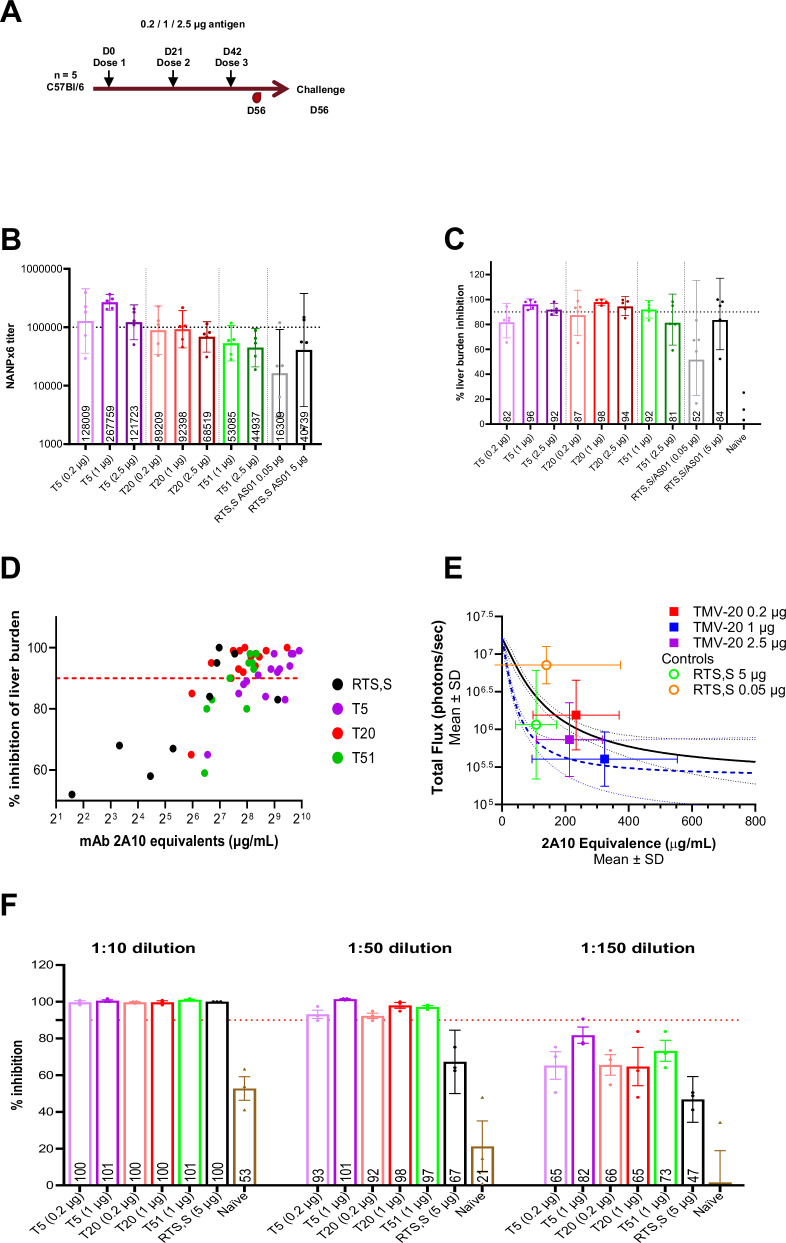
Benchmarking of ALFQ-adjuvanted junctional+minor repeat and major repeat TMV constructs against RTS,S/AS01 in the Pb-PfCSP in vivo liver burden model (JHU mouse study 1). **A** Schematic of study outline. C57Bl/6 mice (*n* = 5) were vaccinated with ALFQ-adjuvanted T5, T20 (0.2-, 1, or 2.5 µg dose), T51 (1 or 2.5 µg dose), or RTS,S/AS01 (0.05 or 5 µg dose). Sera was collected at 2WP3, and mice were challenged via intravenous administration of 2000 *Pb-Pf*CSP transgenic sporozoites. The liver burden reduction was assessed 42 hours post-challenge. **B** NANPx6 geometric mean ELISA titers with 95% confidence intervals of T5-, T20-, and T51-vaccinated sera for each dose group as compared to RTS,S/AS01. **C** Geometric mean percentage liver burden inhibition with 95% confidence intervals of T5-, T20-, and T51-vaccinated mice sera for each dose group as compared to RTS,S/AS01 (luminescence signal compared to naïve controls). **D** Inhibition of parasite burden *vs*. 2A10 equivalents for individual mice. Points are colored according to the dose group schema used in (**B**) and (**C**). **E** Modeled liver burden (total flux) *vs*. antibody concentration (mAb 2A10 equivalents). The black solid line represents historic RTS,S/AS01 vaccine performance in the JHU model, and the dashed blue line represents a theoretical 3x improvement over RTS,S/AS01. **F** ILSDA results for select ALFQ-adjuvanted vaccine groups conducted at 1:10, 1:50, and 1:150 serum diluions. Mean values with 95% confidence intervals are plotted.

Prioritizing in vivo protection in the WRAIR challenge model over in vitro neutralization data, we evaluated three protective vaccines in the JHU model: T51 (junctional+minor repeat), T5, and T20 (major repeat). C57Bl/6 mice (*n* = 5) received three immunizations of ALFQ-adjuvanted T5 (0.2, 1, or 2.5 µg), T20 (0.2, 1, or 2.5 µg), or T51 (1 or 2.5 µg), or AS01-adjuvanted RTS,S (0.05 or 5 µg) (Fig. 4A). The immunogenicity of individual dose groups and combined dose groups were compared using a NANPx6 ELISA, which indicated that T20 and T51 were similarly immunogenic to RTS,S (5 µg), while T5 was significantly more immunogenic (Fig. 4B and Supplementary Fig. 3A). Parasite liver burden quantified 2 days after intravenous challenge with luciferase-tagged *Pb*-*Pf*CSP parasites revealed >90% inhibition was affected across T5- and T20-vaccinated groups, with no significant differences between the T5, T20, and T51 groups and RTS,S (5 µg) (Fig. 4C and Supplementary Fig. 3B). The inhibition of parasite burden *vs*. total circulating anti-CSP antibodies (as measured by mAb 2A10 equivalents) showed that relatively high CSP titers were associated with >90% inhibition for T5, while for T20, T51, and RTS,S, this level of inhibition was achieved at lower titers (Fig. 4D). Modeling parasite burden (total flux) *vs*. mAb 2A10 equivalents for historical RTS,S/AS01 trials in the JHU model (black solid line, Fig. 4E)^49^ revealed that T20 at a 1 µg dose approached the inhibition profile of a hypothetical vaccine candidate with a 3x improvement over RTS,S/AS01 (blue dotted line, Fig. 4E); across all doses, T51 and T5 vaccines did not meet this criterion (Supplementary Figs. 4A and 4B, respectively).

ILSDA on individual mouse serum from the first JHU study showed >90% inhibitory activity across all vaccine groups at a 1:10 dilution (Fig. 4F). However, at higher dilutions (1:50 and 1:150), RTS,S recipients showed lower inhibition than TMV recipients. The first JHU mouse study was used to down-select the T20 construct for process development. A subsequent study (JHU Study 2) compared three doses of a cGMP-compliant T20/ALFQ batch (1 or 2.5 µg), T5/ALFQ (1 or 2.5 µg), and RTS,S/AS01 (5 µg). Two weeks following the final vaccination, mice were challenged via five bites from infected *Anopheles stephensi* mosquitoes. The immunogenicity and liver burden inhibition profiles of the cGMP-compliant batch of T20/ALFQ and lab-grade T5/ALFQ in the JHU study 2 were similar to those of RTS,S/AS01 (Supplementary Fig. 5).

### Serological mapping of antibody responses

**Fig. 5:**
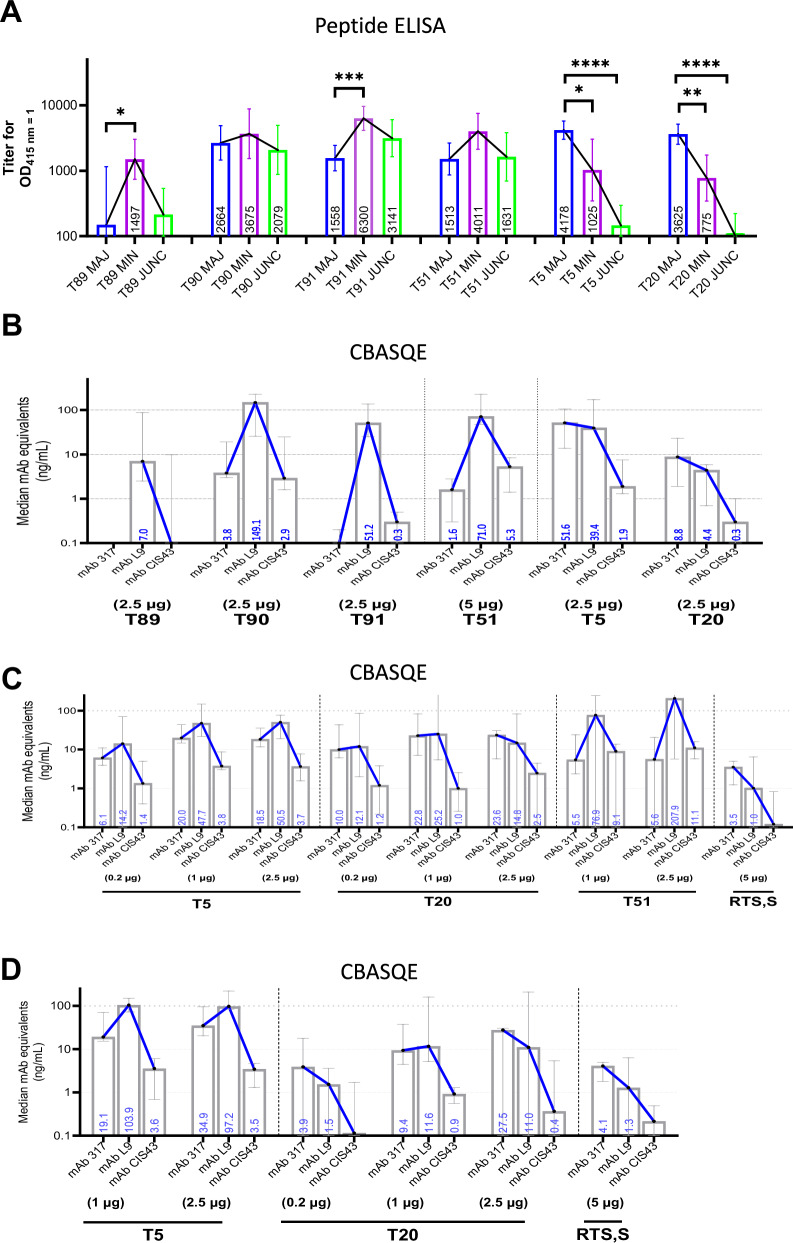
Epitope mapping and CBASQE quantitation of functional binding for 2WP3 vaccine sera. **A** Geometric mean ELISA titer with 95% confidence intervals of Addavax-adjuvanted first screened against using peptides NANPx6 (major repeat, abbreviated as MAJ), peptide 22 (minor repeat, abbreviated as MIN) and peptide 21 (junctional, abbreviated as JUNC). **B** CBASQE-based quantitation of mAb 317, mAb CIS43, and mAb L9 competition (median ng/mL with 95% confidence intervals) for Addavax-adjuvanted vaccines. **C** CBASQE assay using select sera from JHU study 1 showing comparison of TMV vaccines to RTS,S/AS01. **D** CBASQE assay using sera from JHU study 2 showing comparison of T5 and cGMP T20 vaccines to RTS,S/AS01.

To fine-map cross-reactivity profiles, sera from Addavax adjuvanted studies were analyzed by ELISA using peptide 21 (junctional, abbreviated as JUNC), peptide 22 (minor repeat, abbreviated as MIN), and NANPx6 (major repeat, abbreviated as MAJ)^31^. Three serum cross-reactivity profiles were observed: a minor repeat-focused profile (MIN ≈ JUNC > MAJ) for T89 and T91, a cross-reactive profile (MIN ≈ JUNC ≈ MAJ) for T90 and T51, and a major repeat-focused profile (MAJ > MIN > JUNC) for T5 and T20 (Fig. 5A).

A novel CSP-based assay for serological quantification and equivalency (CBASQE) assay was used to measure the ability of polyclonal antibodies to outcompete the binding of the protective CSP mAbs 317, L9, and CIS43 to their cognate epitopes (reported as ng/mL mAb equivalents)^52^ (Fig. 5B–D). Addavax-adjuvanted TMV vaccines showed two mAb competition profiles: a minor repeat-focused profile (L9 > CIS43 ≈ 317) shared by T89, T90, T91, and T51, and a major repeat-focused profile (317 ≈ L9 > CIS43) shared by T5 and T20 (Fig. 5B). CBASQE using sera from the JHU studies 1 and 2, where TMV vaccines were tested with the ALFQ adjuvant, showed that all TMV vaccines had higher ng/mL mAb equivalents compared to RTS,S/AS01 (Fig. 5C, D). Overall, two CBSAQE profiles were observed: a minor repeat-focused profile (L9 > CIS43 ≈ 317) for T51 and a major repeat-focused profile (317 ≈ L9 > CIS43) for T5, T20, and RTS,S (Fig. 5C, D). A key difference between T5 and T20, observed in both JHU studies, was the greater L9:317 ng/mL ratio for T5 antibodies.

## Discussion

Designing new malaria vaccines that recapitulate the protective effects of neutralizing mAbs requires iterative cycles of VLP design and testing in preclinical murine models. T89, which displayed the core residues contacted by mAb L9, elicited minor repeat-focused antibodies whose signature closely mimicked the reactivity pattern of mAb L9. However, T89 was poorly immunogenic, likely due to its short, overly constrained epitope (Table 1). Although the particles used in the study were produced using similar purification and refolding protocols, fine structural differences in particles may have also impacted their immunological performance. Nonetheless, constructs with longer versions of the minor repeat epitope (T90 and T91) were immunogenic, and their antibodies efficiently bound sporozoites and outcompeted mAb L9 in the CBASQE assay; however, these antibodies did not affect significant sterile protection in the WRAIR intravenous challenge model. This observed lack of protection does not align with the reported potent efficacy of mAb L9 and L9-based vaccine candidates in mice^40,53^. The discrepancy between the protection offered by our vaccine candidates compared to mAb L9’s may simply reflect differences between active immunization and passive administration of mAbs. The latter discrepancy may be attributable to differences in challenge models, as our intravenous challenge model may require antibodies that are predominantly directed against the more abundant major repeat epitope, in contrast to the mosquito bite challenge model used by others. Indeed, anti-CSP mAbs’ cytotoxic activities have been found to be augmented in the skin, an environment where minor repeat- and junctional-focused polyclonal antibodies have been speculated to possess additional cytotoxic activities, as compared to the blood stream^54^.

Despite this poor in vivo protection, high levels of in vitro inhibition were observed for minor repeat candidates. We have previously reported this discrepancy between in vivo and in vitro outcomes when comparing the junctional mAb CIS43 and the major repeat mAb 317^55^. Furthermore, other studies have highlighted that high in vitro activity does not correlate with in vivo efficacy^31,56,57^. This observed discrepancy between in vitro ILSDA and in vivo efficacy for the minor repeat vaccine antibodies remains unresolved. In vivo sterile protection against intravenous transgenic parasite challenge is a highly stringent readout, as infection can be established even if a single sporozoite productively invades the liver. As such, we did not proceed with the minor repeat candidates T90 and T91 despite their ILSDA performance. ILSDA may capture molecular events that only occur when a *P. falciparum* sporozoite invades human liver cells that are not reproduced by transgenic parasites invading mouse liver cells. Higher levels of in vivo protection by mAb L9 or antibodies with similar features (like Ky224) may require the recruitment of rare B-cell lineages and/or specific affinity maturation events to acquire homotypic binding, which may have been difficult to recruit by vaccination^28,37^. Lastly, C57Bl/6 mice lack *VH3-33/30* genes, precluding true mAb L9-like antibody elicitation^29,58^. We did not further explore the causes of this discrepancy, which is a critical limitation of this study. The use of wild-type *P. falciparum* in humanized mice could be considered for future vaccine down-selection studies^56^. Moreover, since we only tested this vaccine in female mice, sex-related differences in response still need to be studied.

Cross-reactivity between major, minor, and junctional epitopes is hypothesized to be an attribute of potently protective mAbs^28^. Despite binding similarly to the major and minor repeats by ELISA, T90 and T91 antibodies showed preferential competition with mAb L9, but not mAb 317, in the CBASQE assay. Overall, a major repeat-focused CBASQE response profile and higher avidities in the major repeat vaccine groups were associated with sterile protection^29^, and minor repeat vaccines, which lack these features, did not improve upon the in vivo protection elicited by major repeat vaccines. These results are in agreement with other recent reports on subdominant epitopes^29,45^. Notably, while this manuscript was under review, Langowski et al.^59^ confirmed our conclusions, with major repeat vaccines being more protective than junctional and non-native minor constructs.

The only non-major construct that demonstrated significant sterile protection in the WRAIR challenge model was the junctional+minor repeat construct, T51. In addition to the minor repeat, T51 encompasses the terminal NPDP and NPNA motifs that are requisite for high-affinity mAb CIS43 and mAb 317 binding^31,46^. The addition of these terminal motifs could also allow for better positioning of the “NPN” core epitope recognized by most inhibitory CSP mAbs, as discussed by Francica et al. (2021)^26,28,29,39^, and likely better resembles native CSP epitope conformations on the sporozoite surface^43^.

T5 was the most immunogenic construct tested, but T5 and T20 were similarly protective in the WRAIR challenge model. The higher immunogencity of T5 was likely due to the reduced flexibility displayed by its constrained loop^60^. In the JHU model, ALFQ-adjuvanted T5 was again more immunogenic than T20 and RTS,S. Transgenic parasite challenge resulted in saturating liver burden reduction, with no apparent significant differences, across vaccines. Further refinement of the challenge model such as using a 2-dose regimen may improve stringency in future studies. The decision to down-select T20 over T5 for cGMP manufacture relied on certain assumptions of antibody quality. T20 had a higher mAb 317:L9 bias in the CBASQE assay (Fig. 5C, D) and achieved equal liver burden reduction at a lower antibody concentration than T5 (Figs. 4D, E, and Supplementary Fig. 5). While the exact mechanism underlying this warrants further investigation, the observed higher protection per unit of T20 antibodies may be due to the formation of more extensive homotypic interactions. CSP on the sporozoites is speculated to be folded into a higher order structure, and it is possible that presentation of extended repeats in a loop conformation accurately recapitulates this architecture^5,61–63^. The arrangement of the major repeat epitope on T20 may induce antibodies with particular Fab-Fab geometries that are different than T5’s and which may be more effective at inducing cytotoxicity or conformational changes in CSP that unmask the C-terminus^30,64^. Further interrogation of the landscape of homotypic interactions may uncover previously unknown attributes of potently protective anti-CSP antibodies, and vaccine candidates such as T5 and T20 may be useful as reagents in identifying these features. We have previously shown T20 to be more protective than T5 at 15 weeks post-third dose in a re-challenge study^44^, and vaccines that protect at lower circulating titers are postulated be more durable. Most importantly, restricted NPNA length, while speculated to elicit higher affinity due to more focused memory B cell responses, does not appear to be significantly more protective than long major repeat vaccines in published studies where 9 *vs*. 27 or 5 *vs*. 20 NPNA vaccines were compared, as discussed by Martin et al. (2023)^30,44,59,65^. Our data corroborates these findings and indicates that a major repeat-dominant response is associated with higher protection in our intravenous challenge models as compared to the higher cross-reactive response induced by junctional and minor repeat candidates. The T20 serological response, which was less cross-reactive than the T5 and had a balanced mAb 317:L9 competition profile, was found to be most optimal.

Approximately 33% of the T20 VLP’s mass is comprised by the major repeat epitope. In contrast, ~16% of the hepatitis B fusion antigen mass used in R21 is the major repeat epitope; since only 1 in 5 monomers are CSP-recombinant in the RTS,S antigen, we estimate ~4% of the RTS,S antigen’s mass is comprised of the major repeats. Thus, T20 tested at a 1 µg dose delivers 0.33 µg of the major NPNA repeats, while RTS,S at 5 µg dose delivers 0.25 µg of the major repeat epitope. We believe that the vaccine doses used in our studies tested nearly equivalent amounts of the repeat epitope. Although several other next-generation CSP vaccines have been recently reported, comparison of protection data across studies is confounded by dose saturation, schedule, number of vaccinations, adjuvants, and the specific transgenic parasite strain used. Head-to-head comparison of these TMV vaccines with other next-generation candidates could be useful in establishing a regulatory pathway for novel CSP vaccines to be tested in the field. Notwithstanding, T20/ALFQ met an a priori established criteria of equivalence to RTS,S based on its favorable antibody quality profile and was selected for cGMP manufacture. With this work, we show that a relatively low-cost *E. coli*-produced VLP can form the basis for a potent next-generation malaria vaccine. The TMV VLP technology can be explored for use in combination vaccines against multiple life stages of *P. falciparum* or vaccines that target multiple *Plasmodium* species.

## Methods

### TMV constructs

Products were purified from *E. coli* as previously described^43^. Briefly, genes encoding each candidate with a N-terminal 6x His tag were cloned into the pD451-SR vector (ATUM, Newark, CA) and transformed into BL21(DE)_3_ *E. coli* cells (Novagen, Madison, WI, USA). Cells were grown at a 1 L-scale in LB media containing 50 µg/mL kanamycin to an OD_600_ value of 0.6–0.8, then induced using 40 µM isopropyl β-D-1-thiogalactopyranoside (IPTG) and incubated overnight at 25 °C with ~200 rpm shaking. Frozen cells were resuspended in 20 mM Tris, 150 mM NaCl (pH 9.0) and lysed using the M-110Y Microfluidizer (Microfluidics, Newton, MA, USA). Urea was added to the lysate to a final concentration of 7 M before centrifugation at 27,000x *g* for 30 minutes at 4 °C. The supernatant was purified over a 5-mL Ni-NTA Superflow resin bed (Qiagen, Germantown, MD, USA) under denaturing conditions, washed using resuspension buffer containing 7 M urea and 20 mM imidazole (5 CV), and eluted with resuspension buffer containing 7 M urea and 250 mM imidazole (5 CV). Eluate fractions containing the product were pooled and passed over a 2-mL Q Sepharose® Fast Flow resin bed for endotoxin removal (Cytiva, Marlborough, MA, USA). The column flow-through was refolded in a Slide-A-Lyzer™ G3 Dialysis Cassette, 10 K MWCO (ThermoFisher Scientific, Waltham, MA, USA) via overnight dialysis at 4 °C against 20 mM Tris, 150 mM NaCl (pH 9.0), 0.05% β-mercaptoethanol. Two additional overnight dialyses occurred at 4 °C against 20 mM Tris, 150 mM NaCl (pH 7 or 7.4, as required by the construct’s pI) for final buffer exchange. Proteins were concentrated as needed to 0.2–0.5 mg/mL using an Amicon® Ultra 10 K MWCO Centrifugal Filter Unit (Merck Millipore, Burlington, MA, USA), filtered through a 0.22 µm PVDF membrane, and stored at -80 °C until use. A publication describing the cGMP-compliant manufacturing process for T20 is currently in preparation.

### Purity and homogeneity

Samples were evaluated for purity on a 4–12% Bis-Tris pre-cast gel (ThermoFisher Scientific) and stained with Coomassie blue. Size exclusion-high pressure liquid chromatography (SEC-HPLC) was performed on a Waters e2695 module (Waters Corporation, Milford, MA, USA) using an Acclaim SEC-1000 4.6 × 300 mm column (ThermoFisher Scientific). 100 μL of sample was analyzed with a flow rate of 0.25 mL/min using 20 mM Tris, 20 mM NaCl (pH 7.0) as the mobile phase. Negative-stain transmission electron microscopy was performed on 300-mesh copper grids (Electron Microscopy Sciences, Hatfield, PA, USA) stained with 2% uranyl acetate and imaged using a JEM-1400 Flash microscope (JEOL, Toyko, Japan) at 30,000x magnification.

### Antigenicity ELISA

A 96-well Immulon™ 4 HBX plate (ThermoFisher Scientific) was coated with 100 ng/well of each TMV construct at 4 °C overnight. Plates were washed with PBS containing 0.01% Polysorbate 20 and blocked with 200 µL/well Blocker™ Casein in PBS (ThermoFisher Scientific). mAbs at 1 µg/mL were serially diluted and incubated on plates for 1 hour, washed, and incubated with 100 µL/well 1:4,000 goat anti-human IgG-HRP (Cat No. 2040-05; Southern Biotech, Birmingham, AL, USA) for 1 hour. Plates were washed, developed for 1 hour with 100 µL/well ABTS^®^ 2-component peroxidase substrate solution (Cat. No. 5120-0033; SeraCare, Milford, MA, USA), arrested with 10 µL/well 20% SDS solution, and read at 415 nm. The concentration of each mAb required for OD_415 nm_ = 1 was reported as antigenicity.

### Mouse study (WRAIR)

Animal studies at WRAIR are performed under an IACUC-approved protocol. Procedures described in this study caused only momentary pain and did not require the use of anesthetics. Female C57Bl/6 mice (The Jackson Laboratory, Bar Harbor, ME, USA) were given three vaccine doses intramuscularly in alternating thighs at 3-week intervals. Antigens were formulated in AddaVax™ (InvivoGen, San Diego, CA, USA) in a 1:1 volumetric ratio. 50 µL of each formulation was administered. Sera was collected from the tail vein 2 weeks after each vaccination. Efficacy was evaluated as described previously via intravenous injection into the caudal vein 3 weeks post-third dose with 100 µL containing 3000 transgenic (*PbPf*) *P. berghei* expressing full-length *P. falciparum* CSP (Wellcome strain)^66,67^. Parasitemia was monitored daily by making a blood smear from a drop of blood collected by tail nick and observing 10–20 fields of a Giemsa-stained blood film by microscopy. Mice were considered positive after 2 consecutive days of parasitemia or negative if no parasitemia was detected up to 2 weeks after challenge. Mice were euthanized by CO_2_ exposure (5 minutes) followed by cervical dislocation per guidelines at WRAIR.

### Mouse Study (JHU)

Mice were under partial anesthesia with isoflurane during blood collection and imaging. WRAIR’s TMV candidates, T5, T20, and T51 (JHU Study 1) and T5, T20, and a cGMP-like batch of T20 (JHU Study 2), were formulated in 50 µL of the Army Liposome Formulation containing QS-21 (ALFQ) adjuvant for evaluation against the RTS,S/AS01 benchmark. RTS,S was administered at either a 0.05 or 5 µg antigen dose^49^ in the AS01_E_ adjuvant diluted 10-fold from the original dose, resulting in 2.5 µg of the TLR4 ligand 3-*O*-desacyl-4’-monophosphoryl lipid A (MPL) and 2.5 µg of the QS-21 saponin. Female C57Bl/6 mice (Charles River Laboratories, Wilmington, MA, USA) were given three vaccine doses intramuscularly in alternating thighs at 3-week intervals. 50 µL of each formulation was administered. Sera collected via retro-orbital bleed two days prior to challenge were used to determine mAb 2A10 equivalencies. Terminal bleeds were also collected. Two weeks after the third dose, protection from parasite liver burden was assessed via intravenous challenge with 2,000 tg*Pb*GFP-Luc*-Pf*CSP parasites (3D7 strain) as described previously^68^. Mice were euthanized by CO_2_ exposure (5 minutes) followed by cervical dislocation per guidelines at JHU. In JHU Study 2, vaccinations were performed as above; however, two weeks following the final vaccination, sera was collected via retro-orbital bleed, and mice were challenged via five bites from infected *Anopheles stephensi* mosquitoes^69^ and evaluated for protection as described above.

### Serological assays

ELISA assays using the plate antigens FL-CSP^70^, NANPx6 peptide, peptide 21 (NPDPNANPNVDPNAN), and peptide 22 (NANPNVDPNANPNVD)^31^ were conducted as described previously^43,44,71^.

### Avidity ELISA

FL-CSP ELISAs were conducted on pooled group sera diluted to its OD_415 nm_ = 1 values as described above, except an additional 30-minute incubation step with a serial dilution of sodium thiocyanate was conducted after the primary incubation step. The avidity index was defined as the thiocyanate concentration required to reduce the OD_415nm_ value to its half-maximum^55^.

### CBASQE assay

CBASQE assays, which assesses the equivalency of vaccine-induced antibodies in relation to well-characterized mAbs targeting key epitopes of CSP, were conducted as described previously^52^.

### Immunofluorescence assay (IFA)

Mosquitoes were dissected in RPMI supplemented with fresh mouse sera, and sporozoites were collected by glass wool filtration. Sporozoites were quantified using a hemocytometer. 10 µL of the sporozoite suspension was applied to 18-well IFA slides and frozen at -80 °C. Slides were thawed, fixed in methanol, and blocked with 1% BSA. 15 µL of various primary serum dilutions were applied for 1 hour in a humidity chamber. Following a PBS wash, 15 µL of 1:5,000 FITC-conjugated secondary antibody (mouse) was applied for 1 hour. After a second wash, slides were imaged under a UV-light microscope at 40x magnification. IFA titer was defined as the highest serum dilution that showed a positive fluorescence signal.

### In vitro liver stage invasion and development assay (ILSDA)

The assay was conducted using mouse sera as described previously^72^.

### Statistical analysis

Data were log-transformed when appropriate, and multiple comparisons were made by an ordinary one-way ANOVA with multiple comparisons by Tukey’s method (GraphPad Prism 10.0 software, La Jolla, CA). For ILSDA data, comparisons were made using a one-way ANOVA with Geisser-Greenhouse correction and multiple comparisons by Tukey’s method. For CBASQE data, comparisons were made using an ordinary one-way ANOVA with multiple comparisons by Tukey’s method. Sterile protection data was assessed by a Mantel-Cox test with Bonferroni’s correction. Statistically significant difference in group means was indicated in figures as **** (*p* < 0.0001), *** (*p* < 0.001), ** (*p* < 0.01), or * (*p* < 0.05).

### Ethics statement

Animal research was conducted under an IACUC-approved animal use protocol in an AAALAC International-accredited facility with a Public Health Services Animal Welfare Assurance and in compliance with the Animal Welfare Act and other federal statutes and regulations relating to laboratory animals.

## Supplementary information

Supplementary Information

## Data Availability

The datasets generated during and/or analyzed during the current study are available from the corresponding author on reasonable request.
