# Supplementary material for: Comparison of major, minor and junctional circumsporozoite protein epitopes for malaria vaccine design
Source: NPJ Vaccines. 2025 Oct 3;10:215. doi: 10.1038/s41541-025-01264-0 (PMC12494720; doi:10.1038/s41541-025-01264-0)

Supplementary Figure 1. ILSDA results using standard CSP mAbs CIS43, 317, and 2A10. Geometric mean values with 95% confidence intervals are plotted.

A

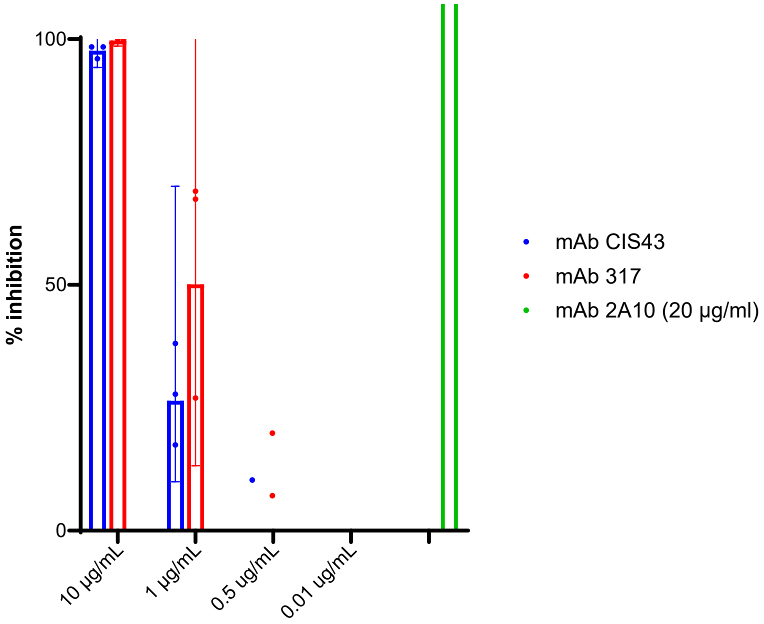

B

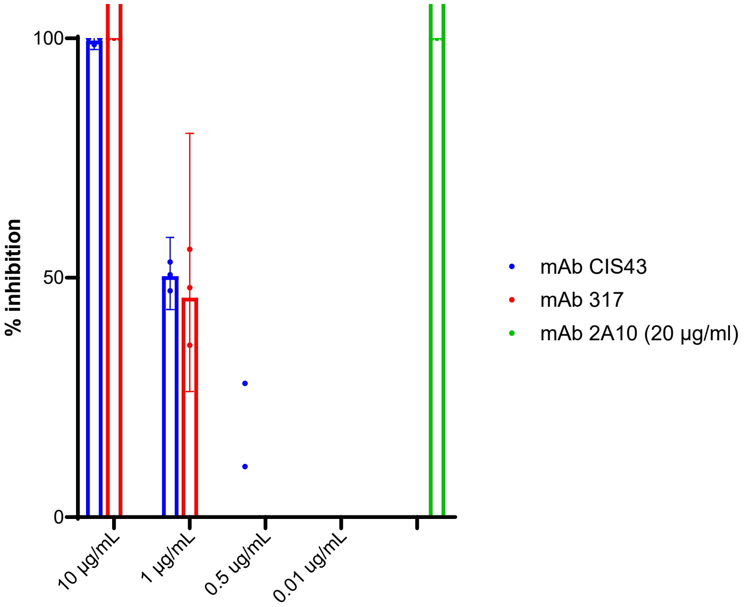

Supplementary Figure 2. The liver burden reduction in the JHU model affected by three vaccinations of FMP013/AFLQ (WRAIR's nearly full-length CSP vaccine) at 10, 5, 2 and 1 µg doses compared to the RTS,S/AS01 vaccine at 0.05 µg, 1 µg and 5 µg doses.

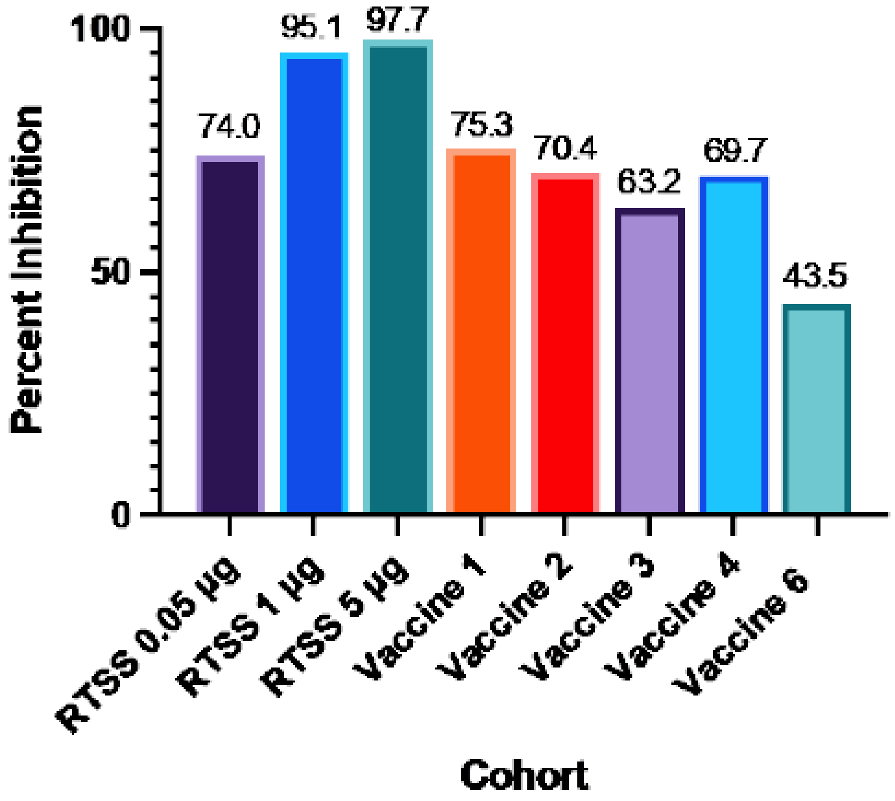

Vaccine 1: FMP013/ALFQ (10 µg)
Vaccine 2: FMP013/ALFQ (5 µg)
Vaccine 3: FMP013/ALFQ (2 µg)
Vaccine 4: FMP013/ALFQ (1 µg)
Vaccine 6: ALFQ alone

Supplementary Figure 3: All dose groups were combined and geometric mean, 95% confidence interval for NANPx6 titer **(A)** and percentage liver burden inhibition **(B),** from JHU mouse study 1 were plotted.

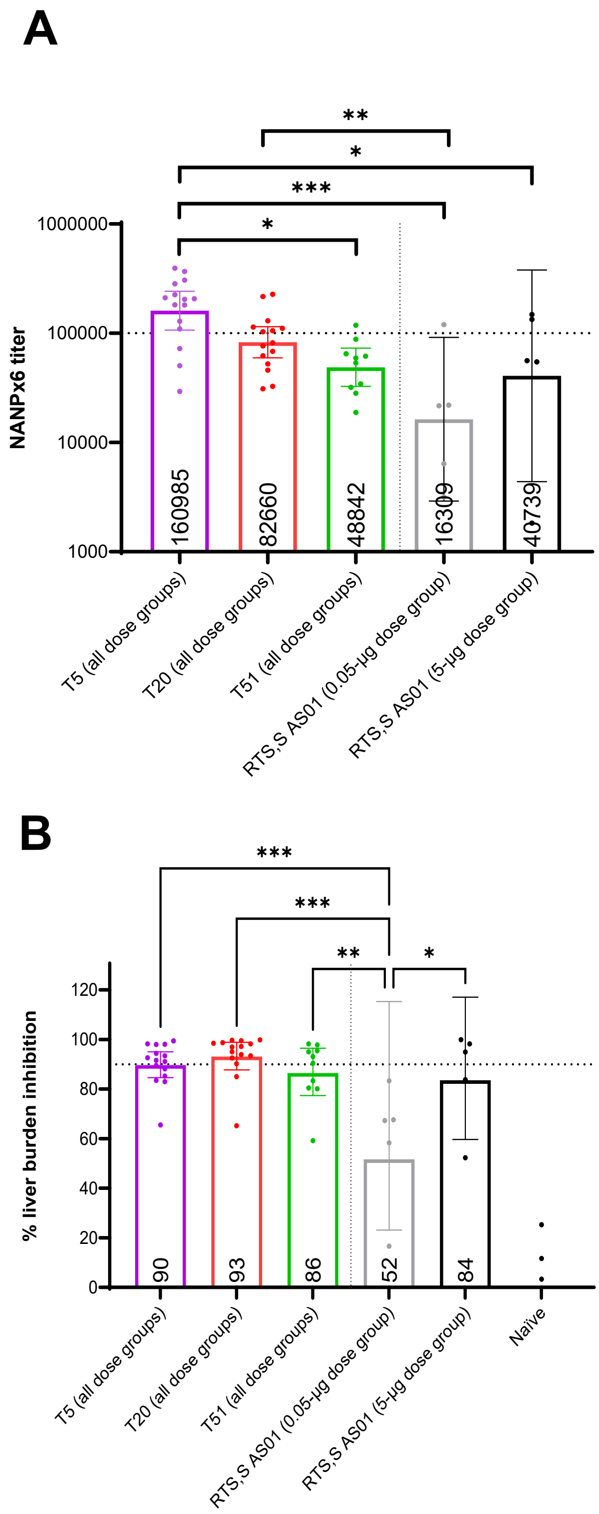

Supplementary Figure 4: Modeling of T51 junctional+minor **(A)** and T5 major repeat **(B)** compared to historical RTS,S/AS01 performance (black line) from JHU mouse study 1. The black solid line represents historic RTS,S/AS01 vaccine performance in the JHU model, and the dashed blue line represents a theoretical 3x improvement over RTS,S/AS01.

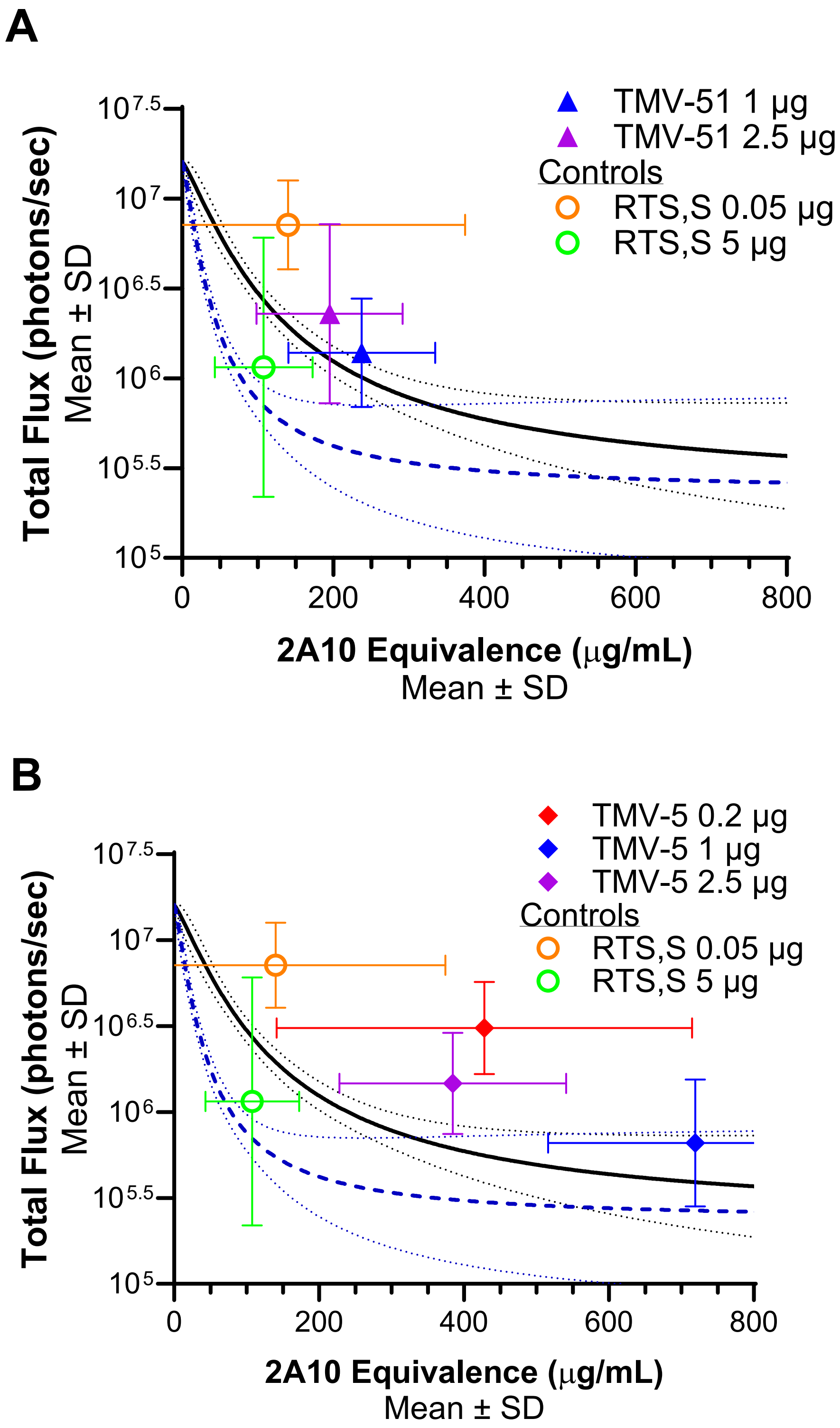

Supplementary Figure 5: The inhibition of parasite burden *vs.* 2A10 equivalents for individual mice vaccinated with T5, T20 and RTS,S (JHU mouse study-2).

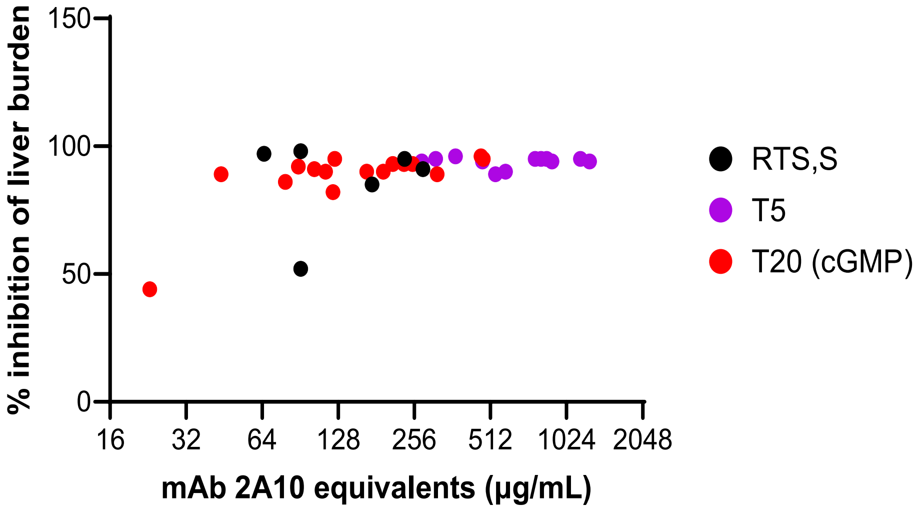

Supplementary Figure 6: Uncropped version of the gel in Fig 1A

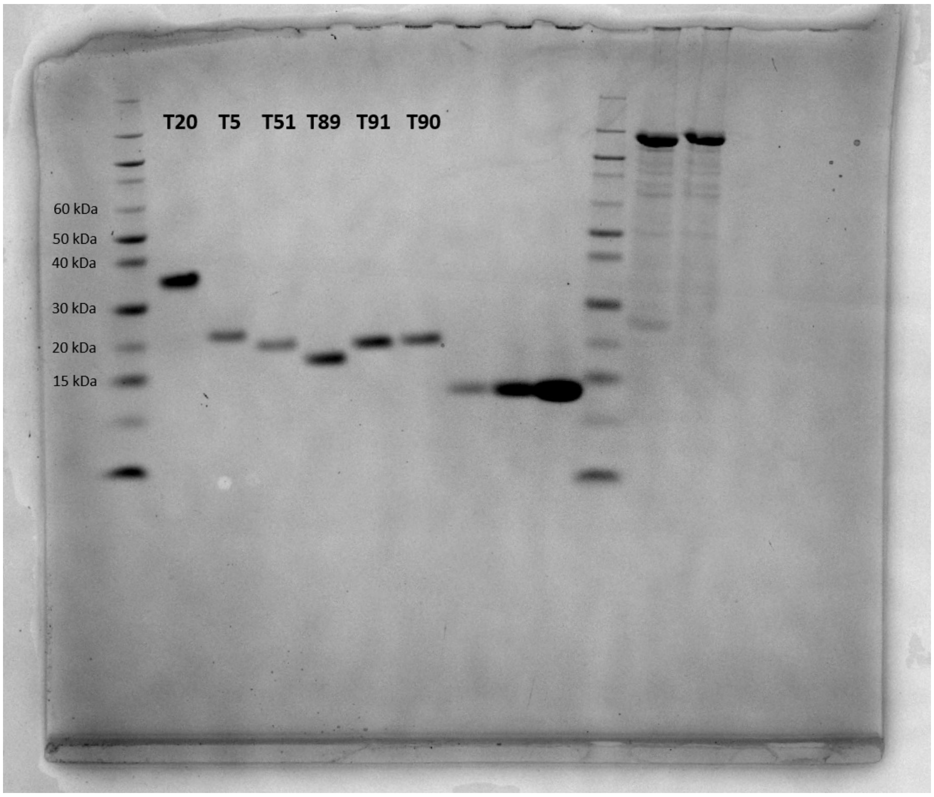

Supplement: Supplementary file 1 — Supplementary Information [file 41541_2025_1264_MOESM1_ESM.pdf]
